# Identification of Glycolysis-Related Genes in MAFLD and Their Immune Infiltration Implications: A Multi-Omics Analysis with Experimental Validation

**DOI:** 10.3390/biomedicines13071636

**Published:** 2025-07-03

**Authors:** Jiawei Chen, Siqi Yang, Diwen Shou, Bo Liu, Shaohan Li, Tongtong Luo, Huiting Chen, Chen Huang, Yongjian Zhou

**Affiliations:** 1 Department of Gastroenterology and Hepatology, Second Affiliated Hospital, School of Medicine, South China University of Technology, Guangzhou 510006, China; 2 Department of Gastroenterology and Hepatology, Guangzhou Digestive Disease Center, Guangzhou First People’s Hospital, Guangzhou 510013, China

**Keywords:** metabolic-associated fatty liver disease (MAFLD), metabolic-associated steatohepatitis (MASH), glycolysis, immune infiltration, bioinformatics analysis

## Abstract

**Background**: Metabolic-associated fatty liver disease (MAFLD) is characterized by metabolic syndrome and immune infiltration, with glycolysis pathway activation emerging as a pivotal contributor. This study aims to identify glycolysis-associated key genes driving MAFLD progression and elucidate their crosstalk with immune infiltration through bioinformatics analysis and experimental validation. **Methods**: Integrative multi-omics analysis was performed on bulk RNA-seq, single-cell RNA-seq, and spatial transcriptomic datasets from MAFLD patients and controls. Differential expression analysis and WGCNA were employed to pinpoint glycolysis-correlated key genes. The relationship with immune infiltration was analyzed using single-cell and spatial transcriptomics technologies. Machine learning was applied to identify feature genes for matching shared TFs and miRNAs. External cohort validation and in vivo experiments (methionine choline-deficient diet murine models) were conducted for biological confirmation. **Results**: Five glycolysis-associated key genes (*ALDH3A1*, *CDK1*, *DEPDC1*, *HKDC1*, *SOX9*) were identified and validated as MAFLD discriminators. Single-cell analysis revealed that the hepatocyte–fibroblast–macrophage axis constitutes the predominant glycolysis-active niche. Spatial transcriptomics showed that *CDK1*, *SOX9*, and *HKDC1* were colocalized with the monocyte-derived macrophage marker *CCR2*. Using four machine learning models, four feature genes were identified, along with their common transcription factors *YY1* and *FOXC1*, and the miRNA “*hsa-miR-590-3p*”. External datasets and experimental validation confirmed that the key genes were upregulated in MAFLD samples. **Conclusions**: In this study, we identified five glycolysis-related key genes in MAFLD and explored their relationship with immune infiltration, providing new insights for diagnosis and metabolism-directed immunomodulation strategies in MAFLD.

## 1. Introduction

Metabolic-associated steatohepatitis (MASH) is the advanced stage of MAFLD (metabolic-associated fatty liver disease), the worldwide incidence of which has reached 30% and is increasing year by year [1]. As a progressive disease, MAFLD/MASH can develop into more severe liver cirrhosis and hepatocellular carcinoma (HCC) [2].

Innate immunity plays a central role in the progression of MAFLD/MASH, with macrophages being a key component of innate immunity. During the progression of MAFLD to MASH, hepatic macrophages are significantly activated, primarily differentiating into M1-type macrophages. Additionally, during the progression of MASH, macrophages undergo metabolic reprogramming, with enhanced glycolysis driving macrophages toward M1 polarization, thereby exacerbating liver inflammation and damage [3]. These macrophages release large amounts of pro-inflammatory factors and reactive oxygen species (ROS), further exacerbating pathological processes such as lipid peroxidation and insulin resistance in the liver, leading to pronounced liver function impairment [4].

MAFLD is strongly associated with features of the metabolic syndrome, including obesity, insulin resistance, and type 2 diabetes (T2D) [5]. A key feature of MAFLD is metabolic dysregulation, with intrahepatic lipid accumulation being strongly linked to insulin resistance and the subsequent disruption of hepatic glucose metabolism [6]. Glycolysis, a critical process in hepatic glucose metabolism, converts glucose into pyruvate and provides energy and intermediates needed for various cellular activities [7]. During the progression of MAFLD, insulin resistance activates the gluconeogenesis pathway, thereby increasing the glucose substrate required for glycolysis [8]. At the same time, mitochondrial dysfunction impairs oxidative phosphorylation capacity, shifting the liver’s energy supply to rely more heavily on the glycolytic pathway [9]. On the other hand, hepatocellular carcinoma utilizes enhanced aerobic glycolysis to support the proliferation, metastasis, and drug resistance of HCC cells [10,11].

Due to the yet unclear pathogenesis, the pharmacological treatment of MAFLD remains challenging. Targeting the glycolytic pathway may represent a feasible approach for treating MAFLD. Studies have shown that the hypoglycemic drug dapagliflozin can improve metabolic indicators, hepatic steatosis, inflammation, and fibrosis in MAFLD mice by downregulating PFKFB3 to inhibit glycolysis [12]. Other research has found that knocking down NOD-like receptor (NLR) X1 (NLRX1) can suppress glycolysis and enhance fatty acid oxidation, thereby reducing hepatic steatosis [13]. However, there is still debate over whether inhibiting the expression of pyruvate kinase muscle 2 (PKM2), a rate-limiting enzyme of glycolysis, can promote Kupffer cell phenotype transformation to alleviate MASH [14,15]. Therefore, targeting glycolysis and identifying key genes represents a promising direction for MAFLD treatment.

In this study, based on the results of differential gene expression analysis and weighted gene co-expression network analysis (WGCNA), we identified five key genes associated with MAFLD and glycolysis. These key genes were further investigated through immune cell infiltration analysis, single-cell sequencing analysis, and spatial transcriptomics analysis. Additionally, four machine learning algorithms were employed to examine these key genes and explore their associated transcription factors and miRNAs. Finally, validation was conducted using external datasets and experimental data. The results indicate that *ALDH3A1*, *CDK1*, *DEPDC1*, *HKDC1*, and *SOX9* are closely related to the glycolytic metabolic pathway in MAFLD, providing new insights into the diagnosis and treatment of MAFLD.

## 2. Materials and Methods

### 2.1. Data Collection and Processing

The bulk RNA-seq datasets GSE213621 (GPL16791), GSE126848 (GPL18573), and GSE33814 (GPL6884), along with the spatial transcriptomics dataset GSE248077 (GPL24247) and the single-cell transcriptomic dataset GSE136103 (GPL20301), were obtained and downloaded from the Gene Expression Omnibus (GEO) database (https://www.ncbi.nlm.nih.gov/geo/, accessed on 5 March 2025). GSE213621 consists of 368 samples: 69 control samples and 299 MAFLD samples. GSE126848 consists of 57 samples: 14 control samples, 12 obese individuals, 15 MAFL samples, and 16 MASH samples, serving as a validation dataset. GSE33814 consists of 44 samples: 13 control samples, 19 MAFL samples, and 12 MASH samples. GSE248077 consists of 6 control samples and 7 MASH samples, and we selected GSM7905572 and GSM7905578 for analysis. In the dataset GSE213621, which is associated with the disease MAFLD, we selected the samples GSM4041156, GSM4041157, GSM4041162, and GSM4041163 for analysis.

The processing of raw datasets was performed using R software (version 4.3.1). Probe ID and gene symbol conversion were performed by connecting to the ENSEMBL database, removing probes without gene symbols, and calculating the average expression value under the same symbol. For high-throughput sequencing data, we used the “DESeq2” package (version1.42.0) for normalization. For the single-cell transcriptomic dataset and the spatial transcriptomic dataset, we applied the “NormalizeData” function and the “SCTransform” function from the “Seurat” package (version 5.0.1), respectively.

The glycolysis genes were obtained from the MsigDB database (https://www.gsea-msigdb.org/gsea/msigdb/index.jsp, accessed on 5 March 2025) by searching with the keyword “glycolysis”. A total of 8 related datasets were retrieved, as shown in Table 1, resulting in a total of 311 glycolysis-related genes.

### 2.2. Differential Gene Expression Analysis

Differential gene expression analysis was performed on the normalized gene matrix using the Wilcoxon rank-sum test. To control for multiple testing, *p*-values were adjusted using the Benjamini–Hochberg method to control the false discovery rate (FDR), while log_2_ fold-change (log_2_FC) represents the base 2 logarithm of the fold change in gene expression between the disease group and the normal group. Differentially expressed genes (DEGs) were selected with thresholds of |log_2_FC|> 0.5 and adjusted *p*-value (adj.P.Val) < 0.05, thereby ensuring both biological relevance and statistical rigor in gene selection. We used the “VennDiagram” R package (version 1.7.3) to find the intersection of DEGs and glycolysis-related genes.

### 2.3. Function Enrichment Analysis

Gene Ontology (GO) and Kyoto Encyclopedia of Genes and Genomes (KEGG) (https://www.kegg.jp/) pathway enrichment analyses were conducted using the “clusterProfiler” R package (version 4.10.0) [16]. Gene Set Enrichment Analysis (GSEA) was also performed using the “clusterProfiler” R package. Based on the log2[Fold Change (FC)] values from the differential analysis, genes were ranked from high to low and defined as the test gene set. Then, the glycolysis-related gene set from the MSigDB database was used to assess whether there were statistical differences in the test gene set.

### 2.4. Implementation of WGCNA and Identification of Key Genes in Key Module

Weighted gene co-expression network analysis (WGCNA) was performed to identify gene modules associated with MAFLD. WGCNA assumes that gene co-expression networks exhibit a scale-free topology [17]. Initially, a gene co-expression similarity matrix was constructed based on pairwise Pearson correlation coefficients between gene expression profiles. This similarity matrix was then transformed into an adjacency matrix using a soft-thresholding power β, which emphasizes strong correlations and suppresses weak ones, aiming to approximate a scale-free network structure. To determine the optimal β value, we evaluated the scale-free topology fit index (R^2^) and mean connectivity across a range of powers, selecting the lowest power at which R^2^ exceeds 0.9 and mean connectivity remains relatively high.

The resulting adjacency matrix was further converted to a topological overlap matrix (TOM) to measure the network interconnectedness, followed by hierarchical clustering to identify modules of highly correlated genes. Dynamic tree cutting was applied to define distinct modules as branches of the clustering dendrogram. Module–trait relationships were assessed by correlating module eigengenes with clinical traits of interest, identifying key modules significantly associated with MAFLD. The key genes were defined as the intersection between genes in these MAFLD-related modules and glycolysis-related differentially expressed genes.

### 2.5. Immune Cell Infiltration Analysis

The “CIBERSORT” R package (version 0.1.0) was used to analyze the proportions of various infiltrating immune cells and whether there are statistically significant differences between the MASH and control groups [18]. Additionally, the Pearson correlation coefficient was calculated to quantify the association between the relative abundance of immune cells and the expression levels of differentially expressed genes.

### 2.6. Single-Cell Transcriptomic Analysis

The “Seurat” R package (version 5.0.1) was used for single-cell sequencing data analysis [19]. During the QC process, both cells with a high percentage of mitochondrial gene counts per cell (>15%) and those with <200 or >5000 identified genes were eliminated. Batch effects were removed using the “Harmony” R package (version 1.2.3). Uniform manifold approximation and projection (UMAP) was applied for dimensionality reduction and clustering. Each cluster was annotated based on marker genes selected from published literature [20]. The “scMetabolism” R package (version 0.2.1) was utilized to assess differences in metabolic pathways between the Control and MAFLD groups, as well as among various cell types [21]. The “CellChat” R package (version 1.6.1) was used to construct intercellular communication networks based on known ligand–receptor interactions [22]. The “AddModuleScore” function was applied to calculate expression scores of the key genes across different cell populations.

### 2.7. Spatial Transcriptomics Analysis

The “seurat” R package (version 5.0.1) was used to create seurat objects and read image information. The “Harmony” R package (version1.2.3) was employed to reduce batch effects between samples. After performing UMAP dimensionality reduction, clustering analysis was conducted using the “FindClusters” and “FindNeighbors” functions. The “FindMarkers” function was used to identify differentially expressed genes between clusters. Clusters were renamed based on markers from published literature, and the expression of each key gene was compared across different clusters [23,24].

### 2.8. Machine Learning

Multiple machine learning (ML) algorithms and combinations were used to train the MASH diagnostic model. External datasets were used for validation, and the algorithms with the highest accuracy, eXtreme Gradient Boosting (XGBoost), Support Vector Machine–Recursive Feature Elimination (SVM-RFE), Least Absolute Shrinkage and Selection Operator (LASSO) regression, and Random Forest (RF), were selected for feature gene screening. The “xgboost” (version 1.7.9.1), “glmnet” (version 4.1-8), “e1071” (version 1.7-16), and “randomForest” R packages (version 4.7-1.2) were used to perform XGBoost, LASSO regression, SVM, and RF analyses, respectively [25,26]. By taking the intersection of the results, four common feature genes were identified.

### 2.9. Gene Regulatory Network

Gene regulatory network analysis comprises two main components: miRNAs and transcription factors (TFs). The 4 common feature genes were used to query the Gene Regulatory Network with NetworkAnalyst (https://www.networkanalyst.ca/home.xhtml, accessed on 25 March 2025). MiRTarBase was used to explore the gene–miRNA interaction networks, and the JASPAR database was used to analyze the TF–gene interaction networks. The degree cutoff was set to 1.0, and the “networkD3” R package (version 0.4) was used to create the Sankey diagram. TargetScan (www.targetscan.org) was utilized to predict and construct the associations between genes and microRNAs.

### 2.10. ROC and Nomogram Model Construction

The “pROC” R package (version 1.18.5) was used to perform multivariable logistic regression analysis on the identified key genes to evaluate their diagnostic significance in MAFLD [27]. Additionally, the area under the receiver operating characteristic (ROC) curve (AUC) was calculated to further assess their predictive accuracy. A nomogram was also developed to predict the probability of MAFLD, along with calibration plots and decision curve analysis to demonstrate the model’s stability. Furthermore, the “edgeR” R package (version 4.0.11) was used to perform gene differential analysis on external datasets, and the area under the ROC curve (AUC) was calculated for the key genes to validate their generalization capability.

### 2.11. Hepatitis Models in Wild-Type Mice

Male C57BL/6 mice (age, eight weeks old) were obtained from Saiye (Suzhou, China) Biotechnology Co., Ltd. These mice were housed in a controlled environment (22 °C with a 12/12 h light/dark cycle) and were provided with water and standard rodent diet. After adaptive feeding, the mice were fed with a methionine choline-deficient diet (MCD) for six weeks to induce MASH. A methionine/choline supplementation (MCS) diet was used in the control groups. At the end of the experiment, liver samples were collected for pathological analysis and transcriptome sequencing. The MAFLD activity scores were used for evaluation, as in a previous publication [28]. All methods were carried out in accordance with relevant guidelines and regulations.

### 2.12. Transcriptome Sequencing

Total RNA was extracted using a Trizol reagent kit (Life technologies, Carlsbad, CA, USA) according to the manufacturer’s protocol. RNA quality was assessed using an Agilent 2100 Bioanalyzer (Agilent Technologies, Palo Alto, CA, USA). The extracted mRNA was enriched using mRNA Capture Beads (Yeasen, Guangzhou, China), and then fragmented by high-temperature treatment. Sequencing libraries were constructed following the protocol of the Hieff NGS^®^ Ultima Dual-mode mRNA Library Prep Kit (Yeasen, Guangzhou, China). PCR amplification was subsequently performed, and final sequencing was carried out using the Illumina NovaSeq X Plus platform at Gene Denovo Biotechnology Co. (Guangzhou, China).

### 2.13. Quantitative Real-Time Polymerase Chain Reaction

Total RNA was extracted from treated cells using Trizol and reverse-transcribed into cDNA (Prime Script RT Reagent Kit (Takara, Otsu, Shiga, Japan)). Then, RT-qPCR was performed using the SYBR^®^ Premix ExTaqTM (Takara, Otsu, Shiga, Japan). GAPDH was selected as the endogenous control gene. Subsequently, the relative gene expression levels were calculated using the comparative Ct method. The primer sequences for the target genes are as follows: *CDK1*: AGGTACTTACGGTGTGGTGTAT(F), CTCGCTTTCAAGTCTGATCTTCT(R); *SOX9*: AGTACCCGCATCTGCACAAC(F), ACGAAGGGTCTCTTCTCGCT(R); *HKDC1*: ACACTTGGTGGCGTTTTACTT(F), CCGCATGTGATACAGGAACC(R); *GAPDH*: ACATCATCCCTGCATCCACT(F), GTCCTCAGTGTAGCCCAAG(R).

### 2.14. Western Blot

Mouse liver tissues were collected and homogenized in WB Tissue/Cell Lysis Buffer (plus protease inhibitor; #AIWB-012, Affinibody, Wuhan, China). After centrifugation (14,000 rpm; 4 °C; 15 min), the supernatant was collected. Protein extracts were quantified using the BCA method and separated on 5–20% SDS-PAGE gels. The separated proteins were transferred onto PVDF membranes and blocked in TBST containing 5% skim milk, after which the proteins were incubated with the primary antibody (diluted 1:1000) at 4 °C overnight. The primary antibodies used are as follows: SOX-9 (# F1113, Selleck, Houston, TX, USA), GAPDH (# A19056, ABclonal, Woburn, MA, USA). After incubation with secondary antibodies (1:5000; #7074; CST, Danvers, MA, USA) at room temperature for one hour, protein visualization was achieved using enhanced chemiluminescence (ECL) detection.

### 2.15. Statistical Analysis

For bioinformatics analysis, we used R language. Experimental data analysis was performed using GraphPad Prism software (version 9.5.0, La Jolla, CA, USA). At least three independent experiments were performed in this study. We compared all data between the two groups using Student’s *t*-test, and we applied the Wilcoxon test for comparisons between multiple groups. Differences were considered to be statistically significant if the *p*-value was less than 0.05. *, *p* < 0.05; **, *p* < 0.01; ***; *p* < 0.001; ****, *p* < 0.0001.

## 3. Results

### 3.1. Identification and Functional Analyses of Glycolysis-Related DEGs

**Figure 1:**
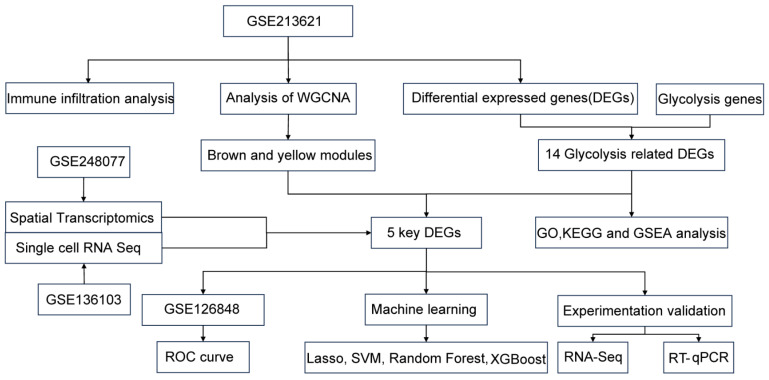
Flowchart of the bioinformatics analysis and experimental validation.

**Figure 2:**
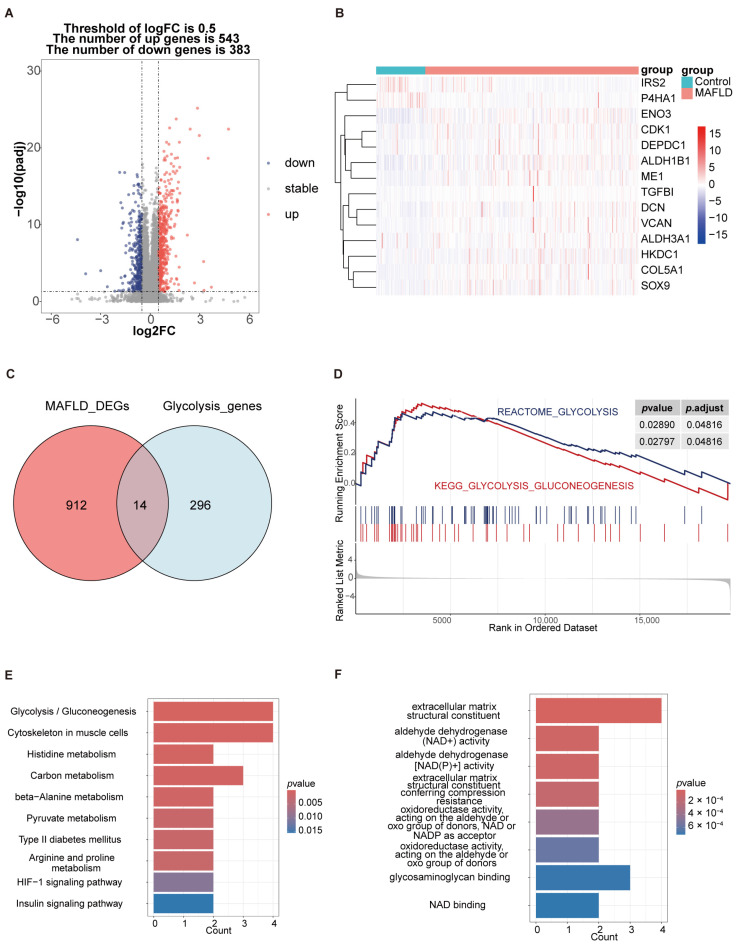
Identification and functional analysis of glycolysis-related differentially expressed genes (DEGs). (**A**) Volcano plot displaying DEGs in liver tissues from MASH patients compared to healthy controls. (**B**) Heatmap showing the expression profiles of glycolysis-related DEGs in healthy controls and MAFLD patients. (**C**) Venn diagram illustrating the intersection of DEGs and glycolysis-related genes. (**D**) GSEA of the glycolysis pathway. (**E**) KEGG pathway enrichment analysis of glycolysis-related DEGs. (**F**) GO pathway enrichment analysis of glycolysis-related DEGs.

Figure 1 was created to illustrate the flowchart of our data analysis process. The analysis of DEGs in the GSE213621 dataset revealed significant expression differences between MAFLD patients and healthy controls, with 543 upregulated genes and 383 downregulated genes (Figure 2A). Among these, 14 DEGs were related to glycolysis, of which 12 were upregulated (Figure 2B,C). GSEA analysis further emphasized that glycolysis metabolism was significantly elevated in MAFLD patients, with an adjusted *p*-value of 0.04816 (Figure 2D). GO analysis showed that these 14 glycolysis-related DEGs were enriched in extracellular matrix structural components and closely associated with aldehyde dehydrogenase (NAD+) activity. KEGG analysis indicated that they were significantly enriched in biological pathways such as glycolysis/gluconeogenesis and carbon metabolism (Figure 2E,F). These findings suggest that in addition to lipid metabolism disorders, MAFLD patients also exhibit abnormalities in the glycolysis metabolic pathway. The close connection between glycolysis and lipid metabolism provides potential therapeutic targets for MAFLD interventions.

### 3.2. Identification of Modules Associated with MAFLD and Glycolysis-Related Key Genes

**Figure 3:**
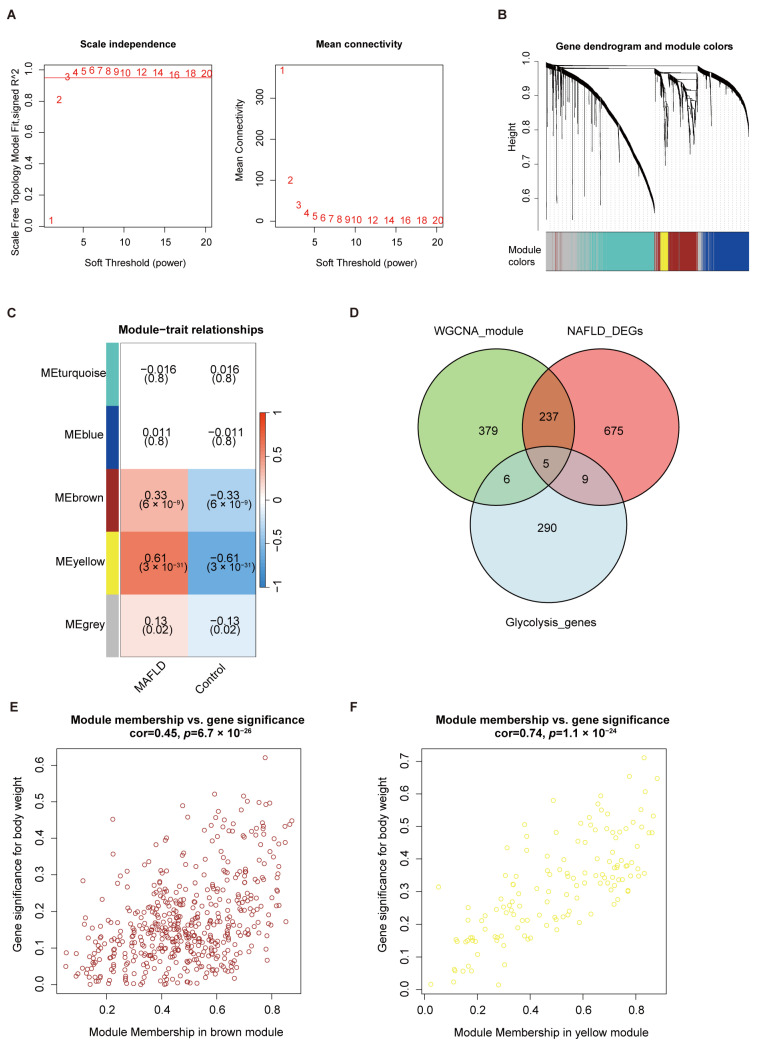
Identification of key modules associated with MAFLD and glycolysis-related key genes. (**A**) Determination of the soft-thresholding power in WGCNA. (**B**) Cluster dendrogram of the WGCNA analysis. (**C**) Module–trait relationship heatmap. (**D**) Venn diagram showing the overlap between key modules and glycolysis-related DEGs. (**E**,**F**) Scatter plots showing the correlation between GS with MM in the brown and yellow modules, respectively.

To identify modules associated with MAFLD, we performed WGCNA analysis on the top 2500 genes with the highest median absolute deviation (MAD). Outlier samples were excluded prior to analysis (Figure S1A). A soft-thresholding power of 3 was selected, as it was the lowest value at which the scale-free topology fit index reached R^2^ ≥ 0.9 while preserving adequate mean connectivity (Figure 3A), thereby ensuring the reliability of the constructed network. Using the dynamic tree-cut algorithm, four independent co-expression gene modules were detected (Figure 3B). These modules were then visualized in a Topological Overlap Matrix (TOM) heatmap (Figure S1B). Then, we evaluated the correlation between these gene modules and MAFLD, revealing a strong correlation between the MEyellow module and MAFLD (correlation coefficient = 0.61), while the MEbrown module showed a moderate correlation (Figure 3C). To further validate this association, we examined Gene Significance (GS) and Module Membership (MM) within the yellow and brown modules, which were found to be highly correlated (correlation coefficient = 0.74, *p* = 1.1 × 10^−24^) and moderately correlated (correlation coefficient = 0.45, *p* = 1.67 × 10^−26^), respectively (Figure 3E,F). This demonstrated the relevance of genes in these two modules to MAFLD. Subsequently, we intersected the genes from the yellow and brown modules with glycolysis-related DEGs and identified five key genes: *ALDH3A1*, *CDK1*, *DEPDC1*, *HKDC1*, and *SOX9* (Figure 3D).

### 3.3. Immune Microenvironment and Immune-Related Functions Analysis

**Figure 4:**
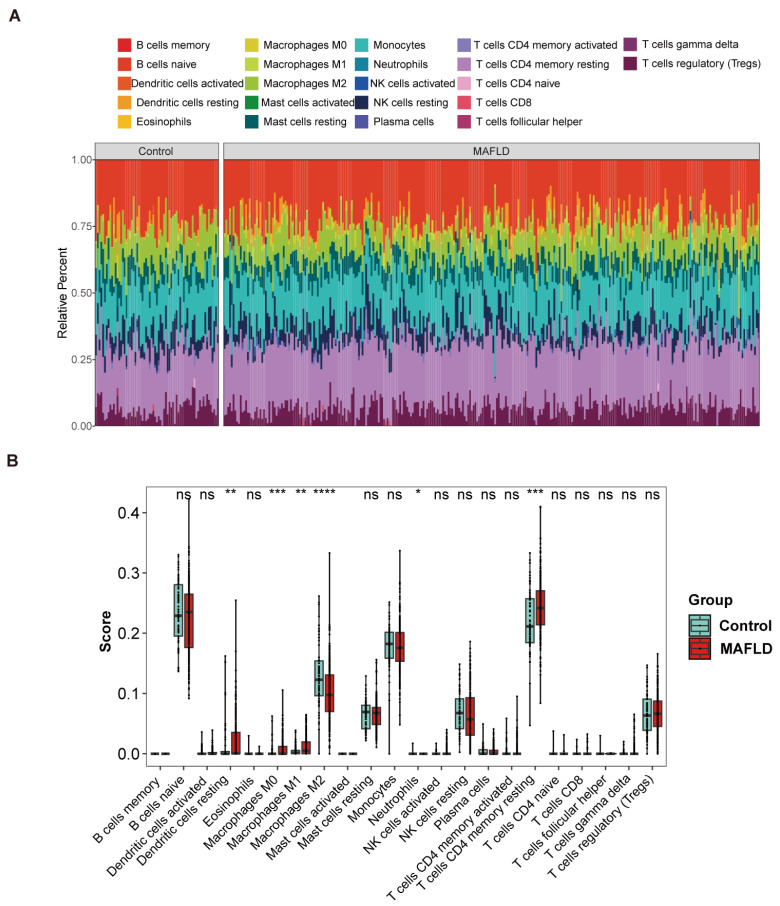

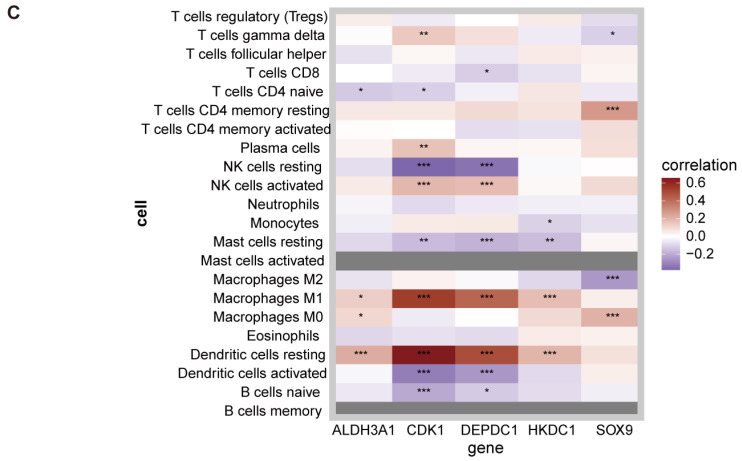
Analysis of the immune microenvironment and immune-related functions. (**A**) Stacked histogram showing changes in immune cell proportions. (**B**) Comparison of immune cell infiltration between the control and MAFLD groups. (**C**) Correlation between infiltrating immune cells and key DEGs. *, *p* < 0.05; **, *p* < 0.01; ***, *p* < 0.001; ****, *p* < 0.0001; ns: not significant.

To investigate the immune response mechanisms in MAFLD, we applied the CIBERSORT algorithm to assess changes in immune cell abundance between MAFLD patients and healthy individuals (Figure 4A). Our results indicate that, compared to controls, the presence of macrophages M0, macrophages M1, T cells CD4 memory resting, and dendritic cells resting significantly increased in NAFLD samples. Conversely, the levels of macrophages M2 were significantly reduced in NAFLD samples compared to the control group (Figure 4B). Additionally, we used a correlation heatmap to illustrate the association between the key genes and various immune cells. *ALDH3A1*, *CDK1*, *DEPDC1*, and *HKDC1* were positively correlated with the levels of macrophages M1, while *SOX9* was negatively correlated with the levels of macrophages M2 (Figure 4C).

### 3.4. The Glycolytic Metabolic Pathway in the Characteristics of Single-Cell Transcriptomics

**Figure 5:**
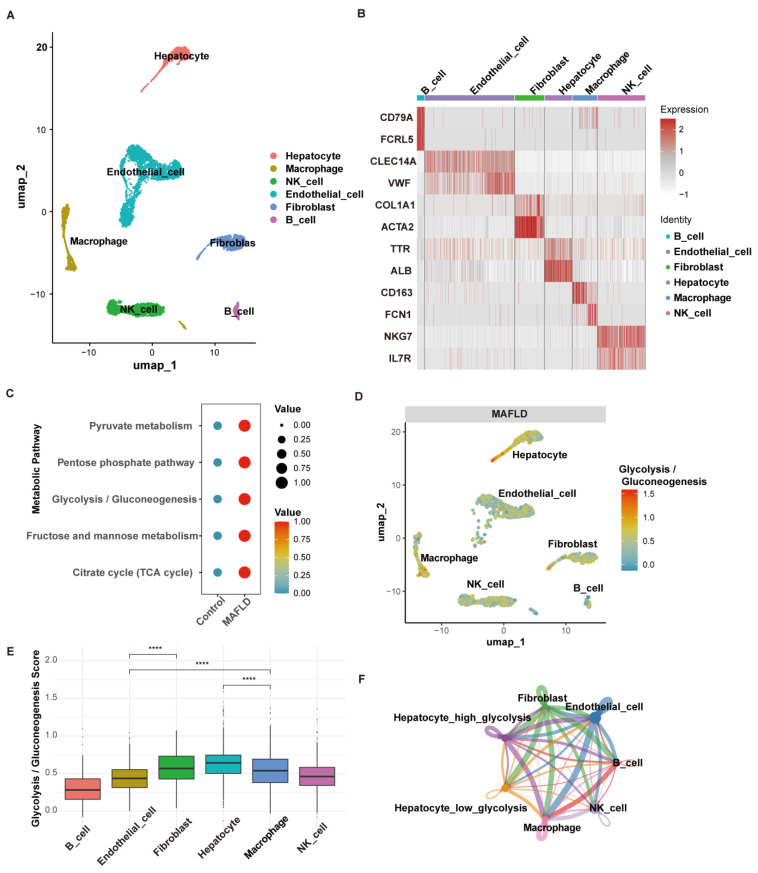
The glycolytic metabolic pathway in the characteristics of single-cell transcriptomics. (**A**) UMAP plot illustrating cell type distribution based on canonical marker genes. (**B**) Heatmap displaying the top two marker genes for each identified cell cluster. (**C**) Dot plot demonstrating significant differences in glycolysis-related metabolic pathway activity between control and MAFLD groups. (**D**) UMAP plot showing glycolysis/gluconeogenesis pathway activity across various cell types in MAFLD samples. (**E**) Box plot depicting glycolysis/gluconeogenesis activity levels among different cell types. (**F**) Circle plot indicating the number of intercellular interactions between immune cells and hepatocytes in high-glycolysis versus low-glycolysis groups. ****, *p* < 0.0001.

Single-cell RNA sequencing (scRNA-seq) was utilized to investigate a liver dataset (GSE136103) to explore the activation of the glycolysis pathway in different liver cell types, including hepatocytes, endothelial cells, and immune cells, in MAFLD. Following quality control, normalization, and batch effect removal, we performed UMAP dimensionality reduction using the top 1 to 10 principal components and selected a resolution of 0.1 for clustering (Figure 5A). Based on specific marker genes corresponding to various cell types from published literature, we identified and annotated six cell types: hepatocytes, endothelial cells, fibroblasts, macrophages, NK cells, and B cells (Figure 5B). To evaluate the differences in glycolytic metabolic pathways between MAFLD and the control group, we used the “scMetabolism” R package to analyze pathway enrichment. The analysis revealed that glycolysis-related pathways were significantly enriched in MAFLD, primarily in hepatocytes and fibroblasts, with macrophages also exhibiting relatively high metabolic activity (adjusted *p*-value < 0.0001, Figure 5C–E). To investigate the liver microenvironment in MAFLD patients, we performed intercellular communication analysis using the “CellChat” R package. Hepatocytes were divided into high-glycolysis and low-glycolysis groups based on glycolytic pathway activity scores. The high-glycolysis group exhibited enhanced interaction pathways with fibroblasts, macrophages, and other immune cells (Figure 5F). Additionally, we applied the “AddModuleScore” function to calculate feature scores for each cell based on the key genes. The results indicated that the key genes were upregulated in MAFLD and were predominantly enriched in hepatocytes. (Figure S2A,B).

### 3.5. Spatial Co-Localization Analysis of the Key Genes and Monocyte-Derived Macrophages Markers

**Figure 6:**
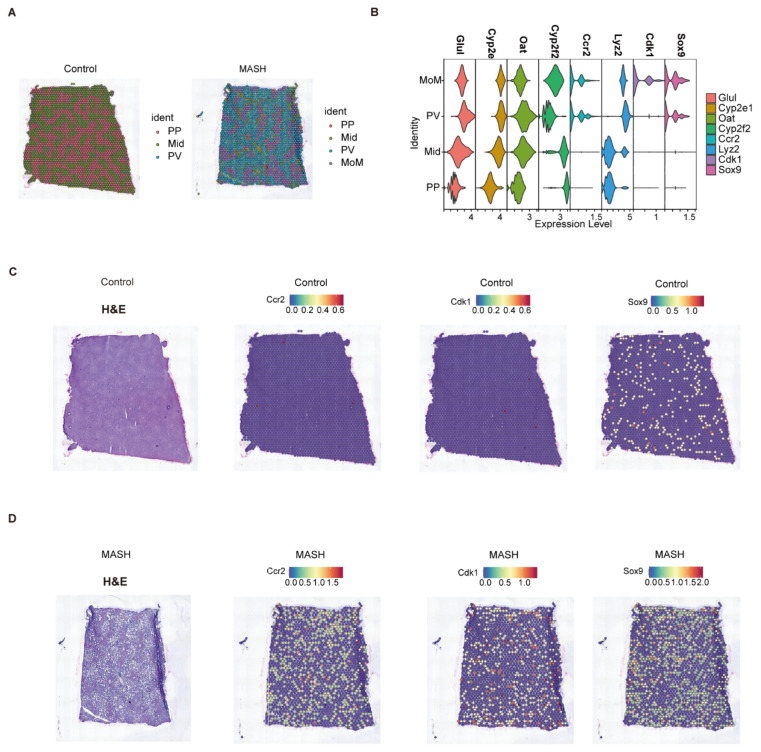
Spatial co-localization analysis of the key genes and monocyte-derived macrophage markers. (**A**) Identified spatial clusters in representative liver sections from MASH and control mice: PV (perivenous zone), PP (periportal zone), Mid (middle zone), and MoM (monocyte-derived macrophage-enriched cluster). (**B**) Violin plots showing the expression levels of markers and the key genes across different zones. *Glul*, *Cyp2e1*, and *Oat* serve as markers for the PP zone; *Cyp2f2* for the PV zone; and *Ccr2* and *Lyz2* for the MoM region. (**C**,**D**) Co-localization of *CDK1* and *SOX9* with the MoM region marker gene *Ccr2*. **Note**: Panels (C,D) show microscope images obtained from GEO database lacking scale or magnification information.

Spatial transcriptomics data GSE248077 were utilized to analyze the distribution of the key genes in liver tissues from MASH and control mice. After normalization, we performed UMAP dimensionality reduction using the top 1 to 20 principal components and selected a resolution of 0.2 for clustering. Based on region-specific markers reported in published studies, we divided liver tissue sections into four zones: periportal zone cluster (PP), perivenous zone cluster (PV), middle zone cluster (Mid), and monocyte-derived macrophage-enriched cluster (MoM), as shown in Figure 6A. To evaluate the enrichment of the key genes in each zone, we plotted violin plots comparing the key genes with marker genes for each zone, as shown in Figure 6B. We observed that *CDK1* was enriched in the MoM region, while SOX9 was enriched in both the MoM and PV regions. Figure 6C,D, along with Figure S2C, illustrate the colocalization of *CDK1*, *SOX9*, and *HKDC1* with the MoM region marker genes *Ccr2* and *Lyz2*, indicating that glycolysis-related key genes are upregulated and enriched in monocyte-derived macrophage-infiltrated regions in MASH.

### 3.6. Identification of Optimal Feature Genes Among Key Genes Using Machine Learning to Construct the TF-mRNA-miRNA Regulatory Mulberry Plot

**Figure 7:**
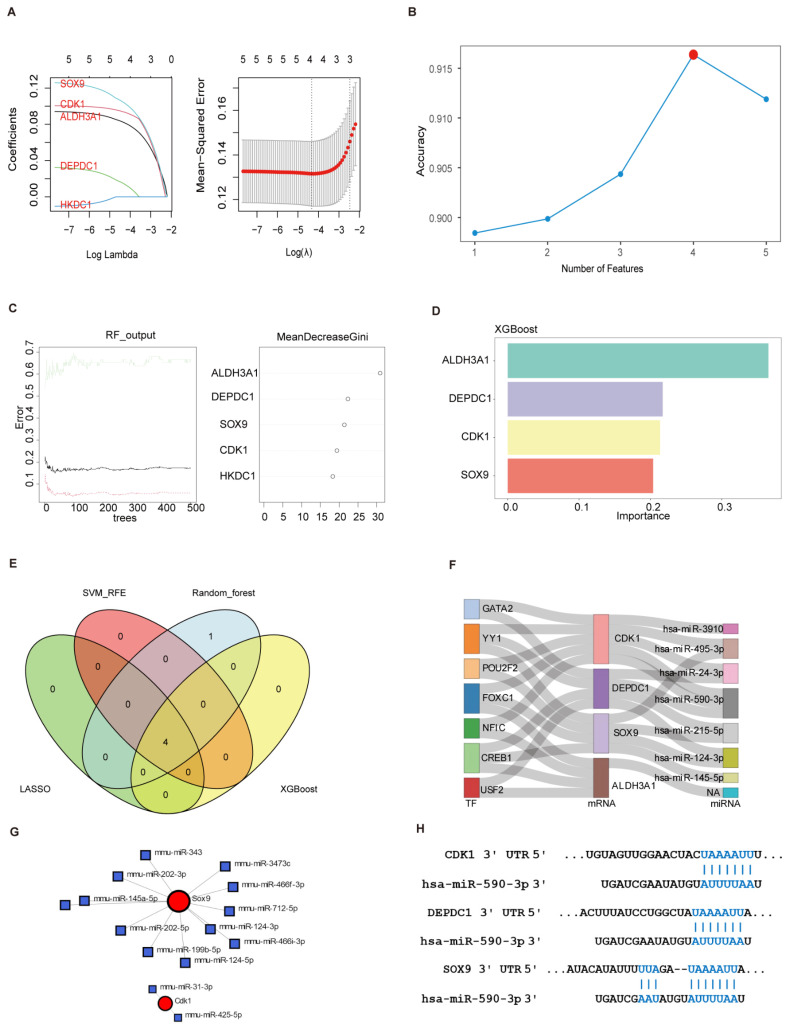
Identification of optimal feature genes among key genes using machine learning to construct the TF–mRNA–miRNA regulatory mulberry plot. (**A**) LASSO regression analysis identified four genes with the lowest binomial deviance as candidate feature genes for MAFLD diagnosis. (**B**) SVM-RFE selected the top four genes based on the highest diagnostic accuracy. (**C**) Random Forest error-rate curves across 0–500 trees (black: overall error; red and green: class-specific errors for the MASH and Control groups, respectively), and gene importance ranked by MeanDecreaseGini. (**D**) Gene importance scores from the XGBoost algorithm. (**E**) Overlap of feature genes Identified by four ML algorithms (**F**) Construction of the TF–mRNA–miRNA regulatory mulberry plot (*H. sapiens*). (**G**) mRNA–miRNA interaction network (*M. musculus*). (**H**) The association between genes and microRNAs.

Four machine learning algorithms were used to identify optimal feature genes based on the five key genes. In the GSE213621 dataset, the LASSO regression algorithm was used to construct a model that minimizes the mean squared error (MSE) and to identify four feature genes: *ALDH3A1*, *CDK1*, *DEPDC1*, and *SOX9* (Figure 7A). The SVM-RFE algorithm, utilizing 10-fold cross-validation, also demonstrated that the model constructed with the same four feature genes achieved an AUC of 0.9163595, indicating that our model can distinguish MAFLD patients from controls with high accuracy (Figure 7B). The RF classifier and XGBoost algorithm ranked the key genes on the importance scale (Figure 7C,D). Finally, *ALDH3A1*, *CDK1*, *DEPDC1*, and *SOX9* genes were identified as optimal feature genes through a Venn diagram analysis (Figure 7E). A TF–mRNA–miRNA regulatory mulberry plot was constructed using these four feature genes. As shown in Figure 7F–H, several TFs and miRNAs, such as *YY1*, *FOXC1*, and *hsa-miR-590-3p*, were identified as potential common regulators of the four feature genes.

### 3.7. Diagnostic Value of Optimal Feature Genes and Validation of the Key Genes

**Figure 8:**
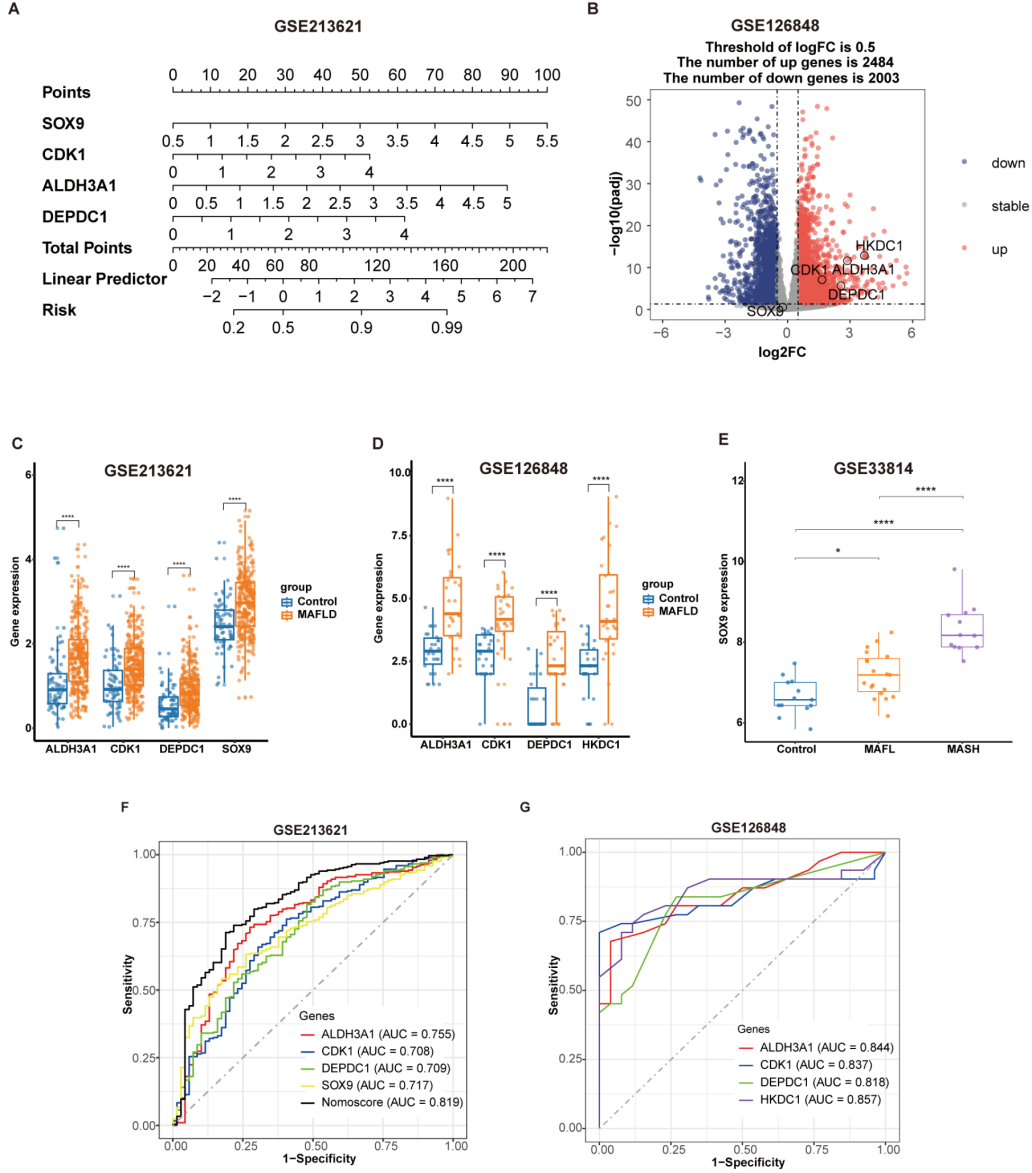
Diagnostic value of optimal feature genes and validation of key genes. (**A**) Nomogram construction using optimal feature genes in dataset GSE213621. (**B**) Volcano plot of the external validation dataset GSE126848, highlighting differential expression of key genes. (**C**,**D**) Expression levels of optimal feature genes in GSE213621 and the four key genes in GSE126848, respectively. ****, *p* < 0.0001. (**E**) Expression levels of SOX9 in GSE33814. *, *p* < 0.05; ****, *p* < 0.0001. (**F**,**G**) ROC curve of key genes in datasets GSE213621 and GSE126848.

In order to determine the accuracy of optimal feature genes in diagnosing MAFLD, we constructed a nomogram using the GSE213621 dataset and analyzed their expression levels and diagnostic performance. The expression levels and diagnostic performance of the four key genes were validated in the external dataset GSE126848. First, a nomoscore chart was constructed based on optimal feature genes where the relative expression of each gene corresponded to a score (Figure 8A). The total score, calculated by summing the scores of each gene, represented the risk score for MAFLD. Figure 8B shows the volcano plot of differential gene analysis in the external dataset GSE126848, where the expression of the four key genes was significantly upregulated, except for *SOX9*, which showed no difference in expression. To further validate this finding, we analyzed the GSE33814 dataset and observed that *SOX9* expression was significantly elevated in MAFLD patients, especially in those with MASH (Figure 8E). Figure 8C and Figure 8D display the expression levels of optimal feature genes in GSE213621 and the key genes in GSE126848, respectively, both of which were significantly elevated in the MAFLD group (*p*-value < 0.0001). The ROC curves in Figure 8F and Figure 8G demonstrate the good predictive performance of the key genes in GSE213621 and GSE126848, respectively, for identifying MAFLD patients.

### 3.8. Experimental Validation of Key Gene Expression in the Mouse MASH Model

**Figure 9:**
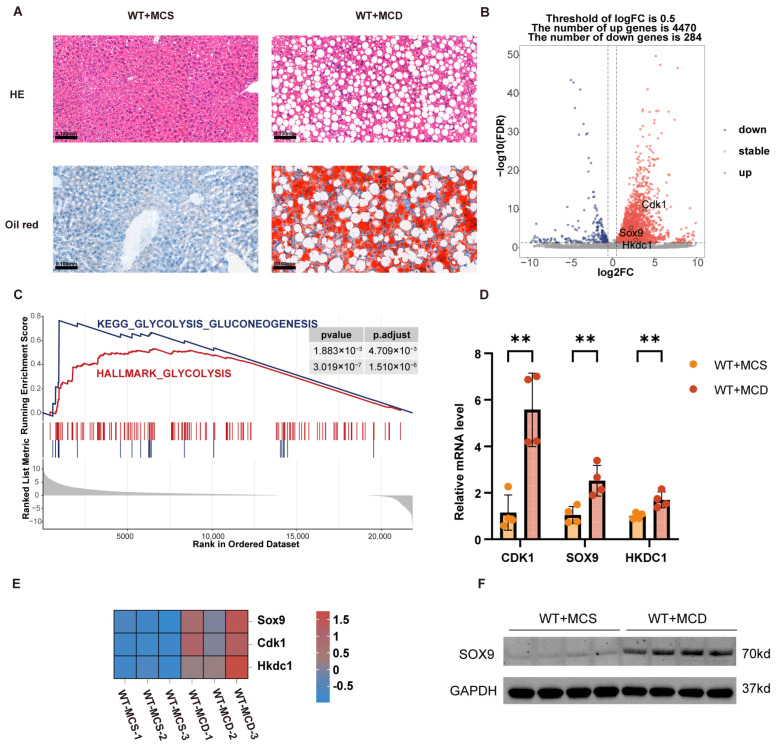
Experimental validation of key gene expression in a mouse MASH model. (**A**) The successful establishment of the MASH mouse model confirmed by HE and Oil Red O staining (n = 4). WT + MCS: Wild-type mice on MCS diet; WT + MCD: Wild-type mice on MCD diet. (**B**) Volcano plot showing differential gene expression between the MASH and control groups, including key genes. (**C**) GSEA reveals enrichment of the glycolysis pathway in MASH model mice. (**D**) Bar chart showing expression differences of *CDK1*, *SOX9*, and *HKDC1* between MASH and control groups. **, *p* < 0.01. (**E**) Heatmap showing differential expression of key genes between MASH and control groups. (**F**) Protein expression levels of *SOX9* in MASH and control groups.

To evaluate the expression levels of the key genes in MASH, we established a MASH mouse model based on WT mice. After six weeks of MCD feeding, severe hepatic steatosis and inflammatory infiltration were observed through HE and Oil Red O staining of liver tissues (Figure 9A). Subsequently, transcriptome sequencing was conducted to examine the expression differences of the key genes between MASH model mice and normal diet mice (Figure S3A). Transcriptome sequencing revealed that the expression levels of the three key genes (*CDK1*, *HKDC1*, and *SOX9*) were upregulated (Figure 9B,E). GSEA analysis indicated significant enrichment of the glycolysis pathway in MASH mice (Figure 9C). RT-qPCR results demonstrated that the transcription levels of *CDK1*, *HKDC1*, and *SOX9* were significantly elevated (Figure 9D). In particular, Western blot analysis confirmed the upregulation of *SOX9* protein expression in MASH (Figure 9F). These findings suggest that these key genes link MASH to glycolysis.

## 4. Discussion

With changes in people’s lifestyles and dietary habits, the incidence of MAFLD has been rising year by year, making it one of the most common liver diseases. It is currently understood that multiple factors—such as insulin resistance due to lipid accumulation and inflammatory infiltration, gut microbiota dysbiosis, and glucolipotoxicity—contribute to the disease’s pathogenesis [29,30]. These factors trigger mechanisms such as mitochondrial dysfunction, endoplasmic reticulum stress, and lipid peroxidation damage, leading to liver inflammation, hepatic stellate cell activation, and, subsequently, the development of MASH, liver fibrosis, and hepatocellular carcinoma [2]. Glycolysis, the initial step in glucose metabolism, not only rapidly supplies energy but also plays a key role in promoting macrophage polarization toward the pro-inflammatory M1 phenotype and supporting cancer cell proliferation [31]. Therefore, investigating the role of glycolytic pathways in MAFLD and immune infiltration may provide valuable insights for therapeutic interventions.

This study analyzed the relationship between MAFLD and glycolysis using datasets from GEO. Through differential gene expression analysis and WGCNA, we identified five key glycolysis-related genes: *ALDH3A1*, *CDK1*, *DEPDC1*, *HKDC1*, and *SOX9*. Immune infiltration analysis revealed significant correlations between these key genes and macrophages. Single-cell RNA sequencing analysis demonstrated that glycolytic activity was predominantly enriched in hepatocytes and fibroblasts, followed by macrophages, within liver tissues from MAFLD patients, highlighting the potential importance of glycolysis in mediating crosstalk among these cell types. Spatial transcriptomics further revealed that *CDK1*, *SOX9*, and *HKDC1* were significantly upregulated in MASH and localized to regions enriched with monocyte-derived macrophages. Using four different machine learning models, we identified four feature genes with diagnostic potential, along with their common transcription factors *YY1* and *FOXC1* and miRNA “*hsa-miR-590-3p*.” Diagnostic utility was evaluated through nomogram construction and ROC curve analysis, confirming the potential of these genes as MAFLD biomarkers. Due to differences in sequencing platforms and cohort heterogeneity, results across different datasets may not be completely consistent. Therefore, we performed supplementary validation using two external datasets and transcriptomic data from a MASH mouse model to ensure the reliability of our conclusions. These results enhance confidence in the identified key genes and establish a foundation for subsequent studies on the contribution of glycolysis to the progression of MAFLD to MASH.

Aldehyde dehydrogenase 3A1 (*ALDH3A1*) is an NAD+-dependent enzyme that oxidizes various endogenous and exogenous aldehydes into carboxylic acids. Previous studies have reported that loss of *ALDH3A1* may lead to an imbalance in glucose homeostasis by impairing energy metabolism [32]. While *ALDH3A1* is expressed at low levels in normal liver tissue, its expression is markedly upregulated in hepatocellular carcinoma (HCC) [33]. These findings suggest that *ALDH3A1* may serve as a potential marker for the transition from MASH to HCC.

Cyclin-dependent kinase 1 (*CDK1*) is a serine/threonine kinase that regulates the progression of the cell cycle from the G2 phase to the M phase and plays a critical role in controlling cell division and glucolipid metabolism [34]. It has been reported that hepatocyte-specific knockout of *CDK1* reduces hepatic triglyceride (TG) levels, but simultaneously impairs fatty acid oxidation (FAO) in hepatocytes, leading to excessive free fatty acids (FFA) that promote hyperinsulinemia [35]. *CDK1* has also been identified as a potential biomarker for HCC [36]. In our study, elevated glycolytic activity in MAFLD was associated with increased *CDK1* expression, suggesting that enhanced glycolysis may contribute to the progression from MASH to HCC via *CDK1*-mediated mechanisms.

DEP domain 1 protein (*DEPDC1*) was initially identified in bladder cancer cells and plays a critical role in the mitotic process [37,38,39]. Recent studies have shown that *DEPDC1* regulates glycolysis in renal cell carcinoma through the AKT/mTOR/HIF1α pathway [39]. Although there is currently no literature directly linking *DEPDC1* to MAFLD, *DEPTOR*, which shares the same DEP domain, is known to activate AKT and glycolytic metabolism [40]. Given that *DEPDC1* plays an important role in both the proliferation and metastasis of HCC [41], investigating its role in the progression of MAFLD holds significant potential value.

Hexokinase domain containing 1 (*HKDC1*), a member of the hexokinase family, acts as a glucose sensor involved in the regulation of glucose metabolism and lipid homeostasis [42]. Previous studies have shown that increased expression of *HKDC1* in MASH contributes to mitochondrial dysfunction in hepatocytes, accompanied by high levels of inflammation and fibrosis [43]. Consistent with these findings, our transcriptomic sequencing data also identify *HKDC1* as a promising biomarker for the progression of MASH.

Sex-determining region Y (SRY)-box 9 (*SOX9*) is a transcription factor belonging to the SOX (SRY-related HMG-box) family [44], which exhibits diametrically opposed effects in MASH and HCC. Although SOX9 expression is significantly elevated in MASH model mice, interestingly, studies have shown that *SOX9* overexpression reduces MASH progression by promoting AMPK activation, while *SOX9* depletion exacerbates MCD-induced changes in lipid metabolism, inflammation, and fibrosis-related pathways [45]. In contrast, previous studies suggest that *SOX9* exacerbates the malignancy of HCC and promotes cancer progression, which may be attributed to *SOX9* being responsible for the induction of *CXCL5* in hepatoma cells, thereby facilitating the proliferation and invasion of HCC tumor cells through autocrine *CXCL5/CXCR2* signaling [46]. At the early stages of liver injury, however, monocyte-derived macrophages rapidly accumulate along the periphery of necrotic areas under the chemotaxis of *CCL2*, inducing a *SOX9*+ hepatocyte-resistant wall that encloses necrotic lesions, thereby protecting undamaged hepatocytes from further injury [47]. This has been confirmed by the colocalization of *SOX9* and *CCR2* observed in our spatial transcriptomics analysis. Furthermore, *SOX9* has been shown to promote M2 macrophage repolarization and inhibit T-cell function [48].

This study has certain limitations. First, both single-cell transcriptomics and spatial transcriptomics technologies inherently have dropout rates, which may lead to the omission of glycolysis genes with low expression levels. Second, the relatively small sample sizes of both the single-cell and spatial cohorts may not fully capture the complexity of MAFLD. Therefore, larger datasets are needed to validate the generalizability of these findings across diverse populations. Finally, further in-depth research is needed to verify the role of glycolysis-related key genes in immune infiltration during the development of MAFLD. In addition, integrating a useful polygenic risk score (PRS) could enhance the prediction of MAFLD, MASH, their associated cirrhosis, and HCC [49].

## 5. Conclusions

In this study, we identified five glycolysis-related key genes (*ALDH3A1*, *CDK1*, *DEPDC1*, *HKDC1*, *SOX9*) associated with MAFLD and determined the activation of the glycolysis pathway in MAFLD as well as its correlation with immune infiltration at both the single-cell and spatial transcriptomics levels. This study provides new insights and perspectives for mechanism research and diagnostic strategies targeting the glycolysis pathway in MAFLD patients.

## Figures and Tables

**Table 1:** Glycolysis-related gene sets and their descriptions in the MSigDB database.

Standard Name	Genes
BIOCARTA_GLYCOLYSIS_PATHWAY	3
KEGG_GLYCOLYSIS_GLUCONEOGENESIS	62
MODULE_306	26
REACTOME_GLYCOLYSIS	74
HALLMARK_GLYCOLYSIS	200
WP_GLYCOLYSIS_IN_SENESCENCE	11
WP_GLYCOLYSIS_AND_GLUCONEOGENESIS	45
WP_AEROBIC_GLYCOLYSIS_AUGMENTED	12

## Data Availability

Transcriptome sequencing data have been uploaded to the SRA database and can be accessed via https://dataview.ncbi.nlm.nih.gov/object/PRJNA1247908?reviewer=2slea82nqcep0k3mvnuog08dvf (accessed on 5 May 2025).

## Supplementary Materials

The following supporting information can be downloaded at: https://www.mdpi.com/article/10.3390/biomedicines13071636/s1, Figure S1: (A) Sample clustering for outlier detection. (B) Topological Overlap Matrix (TOM) heatmap to display the module corresponding to each gene. Figure S2: (A) Comparison of key glycolysis gene activity scores between control and MAFLD groups. (B) UMAP plot illustrating the distribution of key glycolysis gene activity scores across different cell types in MAFLD samples. (C) Co-localization of *HKDC1* with the MoM marker gene *Lyz2*. Figure S3: (A) PCA plot illustrating relationships among transcriptome sequencing samples. (B) Western blot original gel images.

## Abbreviations

The following abbreviations are used in this manuscript:

MAFLD	metabolic-associated fatty liver disease
MASH	metabolic-associated steatohepatitis
HCC	hepatocellular carcinoma
WGCNA	weighted gene co-expression network analysis
DEGs	differentially expressed genes
GO	Gene Ontology
KEGG	Kyoto Encyclopedia of Genes and Genomes
GSEA	Gene Set Enrichment Analysis
UMAP	uniform manifold approximation and projection
XGBoost	eXtreme Gradient Boosting
SVM-RFE	Support Vector Machine–Recursive Feature Elimination
LASSO	Least Absolute Shrinkage and Selection Operator
RF	Random Forest
TFs	transcription factors
MCD	methionine choline-deficient diet
MCS	Methionine/choline supplementation
