# Supplementary figures and images for: Identification of Glycolysis-Related Genes in MAFLD and Their Immune Infiltration Implications: A Multi-Omics Analysis with Experimental Validation
Source: Biomedicines. 2025 Jul 3;13(7):1636. doi: 10.3390/biomedicines13071636 (PMC12293006; doi:10.3390/biomedicines13071636)

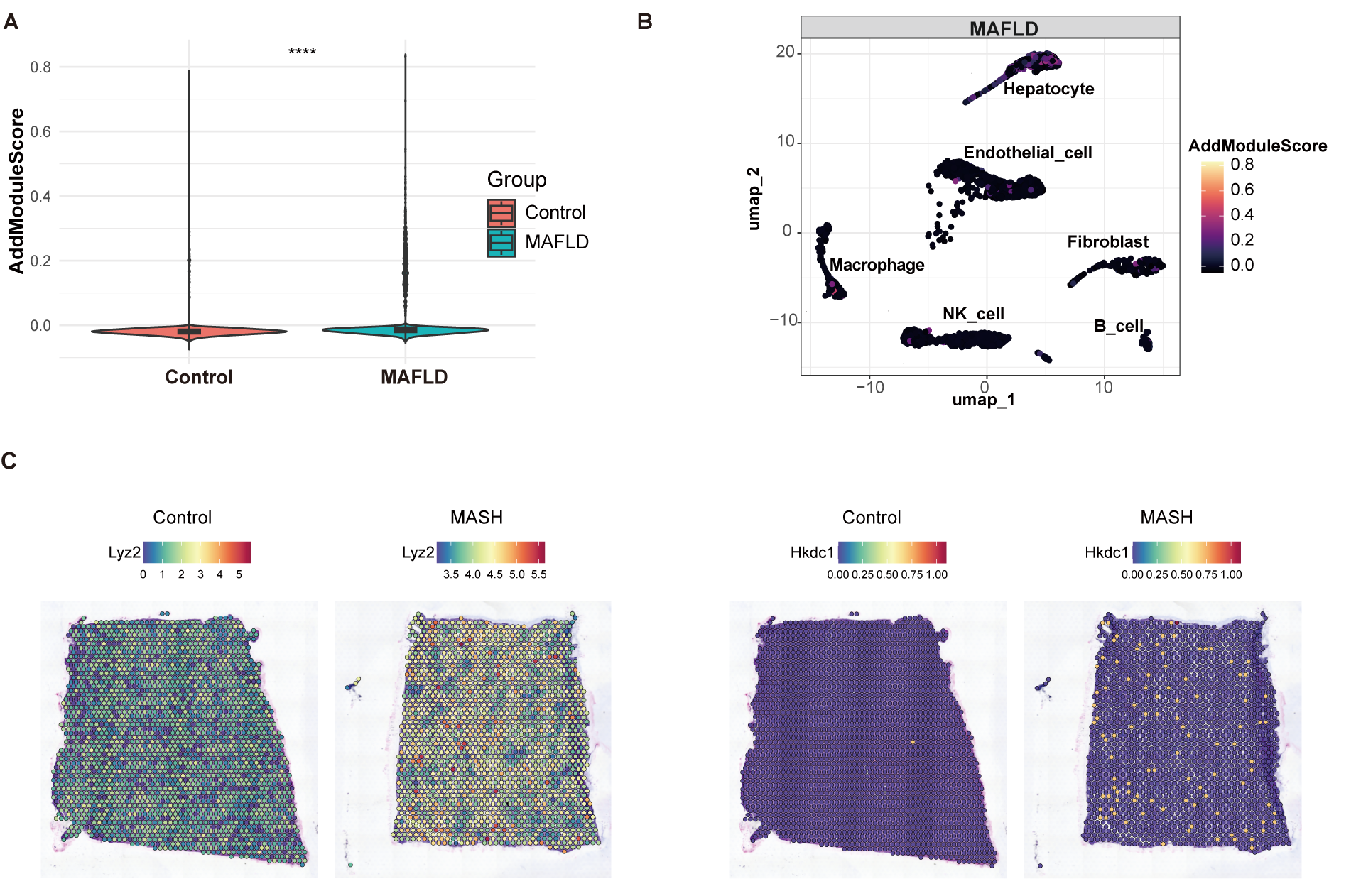

Supplement: Supplementary file 1 [file biomedicines-13-01636-s001.zip › Figure S2.tif]

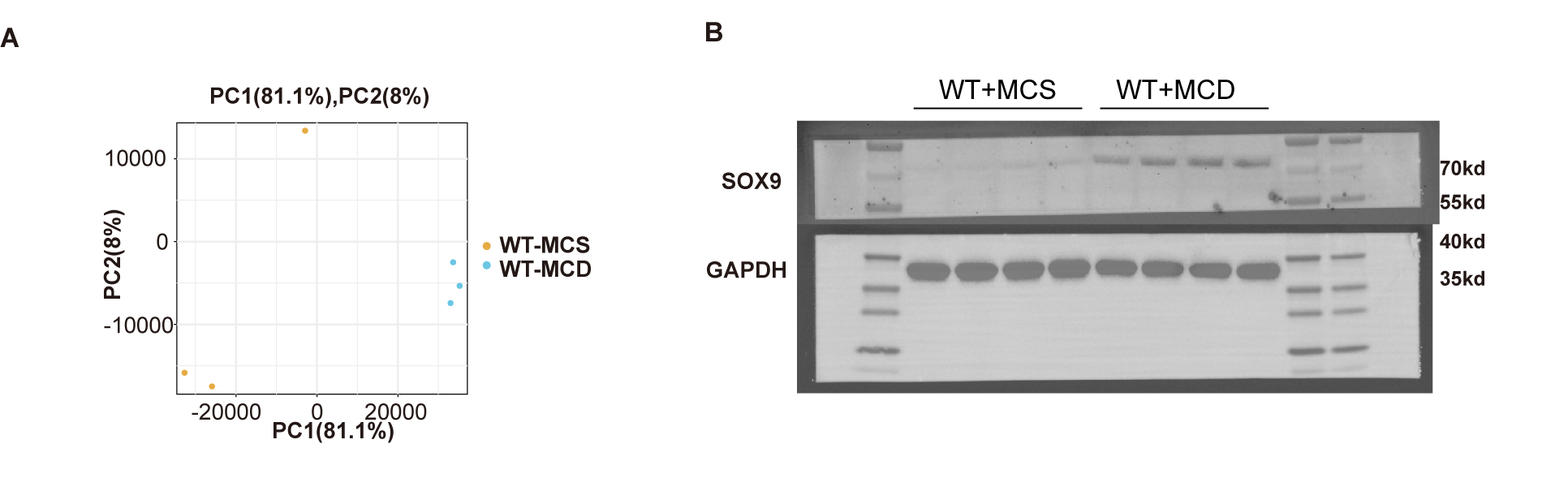

Supplement: Supplementary file 1 [file biomedicines-13-01636-s001.zip › Figure S3.tif]

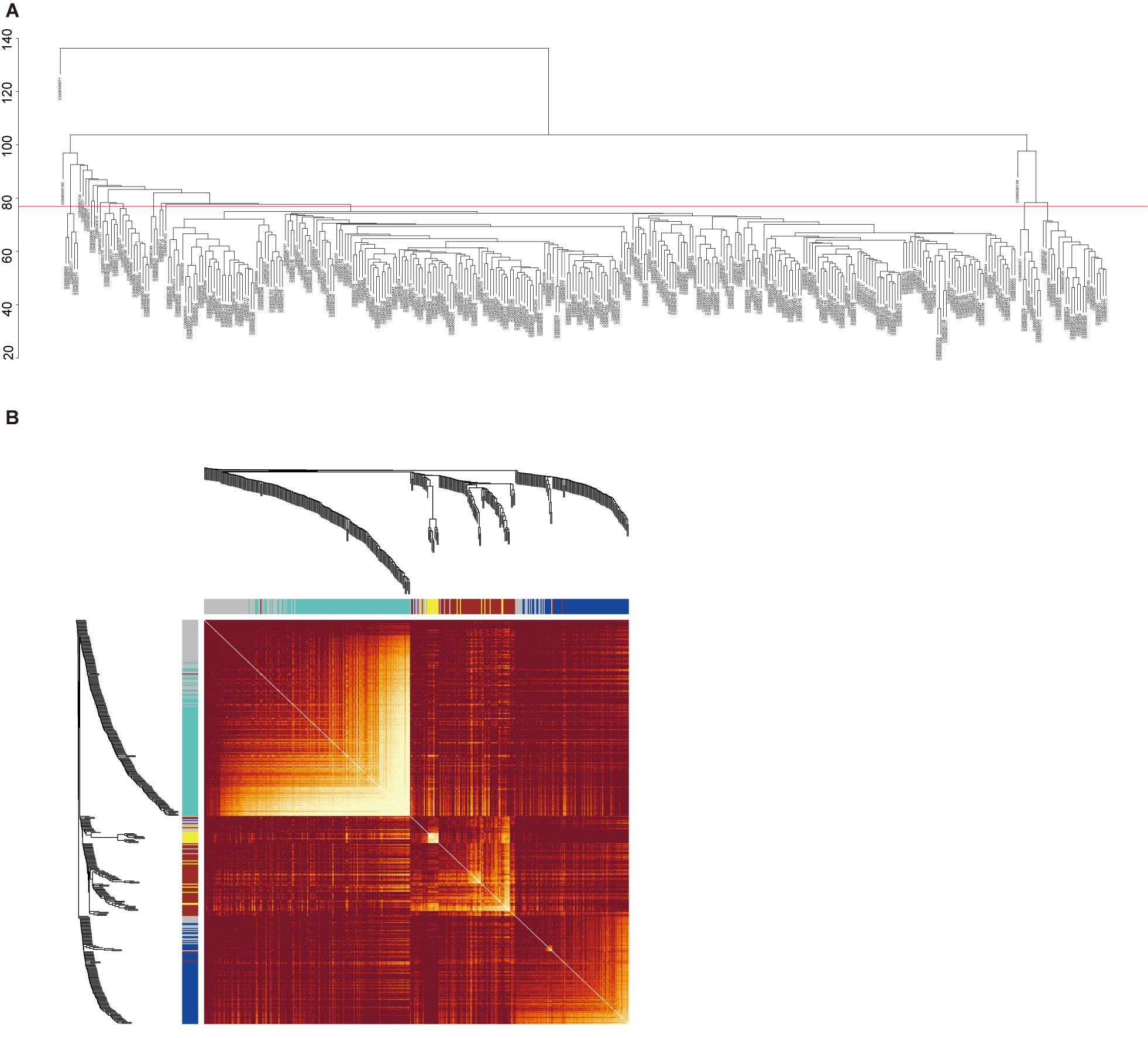

Supplement: Supplementary file 1 [file biomedicines-13-01636-s001.zip › Figure S1.tif]
